# CT-Based Preplanning Allows Abstaining from Intraprocedural TEE during Interventional Closure of the LAA in Patients with Atrial Fibrillation

**DOI:** 10.3390/jcm12124019

**Published:** 2023-06-13

**Authors:** Alexandra Offhaus, Luisa Linss, Peter Roehl, Charlotte Sakriss, Uta Pertschy, Andreas Schwenzky, Henning Ebelt

**Affiliations:** 1 Department of Medicine II, Catholic Hospital “St. Johann Nepomuk”, Haarbergstr. 72, 99097 Erfurt, Germany; 2 Department for Radiology and Imaging, Catholic Hospital “St. Johann Nepomuk”, Haarbergstr. 72, 99097 Erfurt, Germany

**Keywords:** left atrial appendage, atrial fibrillation, LAAC

## Abstract

Objectives: The aim of this study was to determine whether the application of a CT-based preplanning algorithm might allow abstaining from TEE during LAAC. Background: LAAC is an established treatment alternative for patients with atrial fibrillation. Today, most LAAC procedures are guided by TEE, which, however, leads to the need for patient sedation and might even cause direct harm to the patient. CT-based preplanning of the LAAC procedure, in combination with technical improvements in device design and interventional experience, might allow abstaining from TEE. Methods: Fluoro-FLX is a prospective single-center study to evaluate how often TEE leads to a procedural change during interventional LAAC if a dedicated CT planning algorithm is applied. The study hypothesis is that under these circumstances, a sole fluoroscopy-guided LAAC is an alternative to a TEE-guided approach. All procedures are preplanned by cardiac CT and, finally, guided by fluoroscopy only, while TEE is carried out in the background during the intervention for safety reasons. Results: In none of the 31 consecutive patients did TEE lead to a change in the preplanned fluoroscopy-guided LAAC (success ratio: 1.00; CI: 0.94–1.00), thereby meeting the primary endpoint (performance goal: 0.90). There were no procedure-related adverse cardiac or cerebrovascular events (no pericardial effusion, TIA, stroke, systemic embolism, device embolism, death). Conclusions: Our data suggest that it is feasible to perform LAAC under sole fluoroscopic guidance if preplanning is performed using cardiac CT. This might be worth considering, especially in patients who are at high risk for TEE-related adverse events.

## 1. Introduction

Atrial fibrillation (AF), which is the most common cardiac arrhythmia in advanced age, is associated with the risk of intracardiac thrombus formation and the occurrence of stroke and systemic embolism. Oral anticoagulation (OAC) is, nowadays, the standard therapy for patients with AF who have an increased risk of stroke [1]. However, a number of these patients develop bleeding complications that require discontinuation of OAC. In this situation, interventional closure of the left atrial appendage (LAA closure, LAAC) represents an established treatment alternative [2].

In the majority of cases, left atrial appendage closure (LAAC) is performed as a transvenous catheter-based procedure. To enable the correct placement of the occluder, the procedure is usually guided by both angiography (X-ray fluoroscopy) and transesophageal echocardiography (TEE). However, the use of TEE requires either general anesthesia or at least conscious sedation, which are both associated with potential threats and inconveniences to the patient. In addition, TEE, per se, carries the risk of injury to the pharynx and esophagus. In a recent study, it was reported that in patients who underwent a TEE-guided cardiac intervention, lesions in the esophagus caused by TEE could be detected in 86% of all cases with endoscopy [3]. Another study showed that the prolonged use of TEE during structural interventions resulted in clinically relevant complications in 6.1% of cases [4].

Against this background, it seems desirable to be able to perform LAAC without TEE. This is possible, for example, by using intracardiac echocardiography (ICE) instead. The feasibility of ICE-guided LAAC has been shown previously [5], but this method requires a second or enlarged transseptal access and is associated with an increase in procedure-related costs, thereby limiting its clinical use. Alternatively, it also has been described that LAAC might be performed without any ultrasound-based imaging modality by the implementation of 3D rotational angiography [6]. However, this technique is dependent on a temporary “cardiac arrest” through tachypacing and leads to a rise in exposure to X-ray contrast medium and radiation, respectively.

In principle, the use of TEE during LAAC is considered helpful or necessary, especially at certain key steps during the procedure. First, TEE is used to guide transseptal puncture (TSP) to determine the ‘optimal’ puncture site within the interatrial septum, thereby potentially achieving the most favorable access to the LAA, which could facilitate the further course of the procedure. Nevertheless, so far there are no clinical studies that actually have shown an advantage of TEE-guided TSP on the final result of the LAAC procedure with respect to device positioning or completeness of LAA sealing, etc. Second, the use of TEE during TSP is considered to reduce the risk of pericardial tamponade. However, TSP is nowadays performed in daily routine without TEE in a large number of other cardiac interventions (e.g., pulmonary vein isolation, etc.) without a relevant risk of cardiac tamponade so TEE can be considered dispensable in this regard. Third, TEE is used to rule out intracardiac thrombi (not only LAA thrombi but also left atrial cavity thrombi [7]) and to determine the size of the LAA in order to select the suitable size of the LAAC device, although these tasks can be completed already before the implantation procedure, preferably with cardiac CT. Finally, perhaps the most important role of TEE in LAAC is to confirm the correct positioning of the occluder before the device is released definitely. For the WATCHMAN, so-called “PASS criteria” are applied (position, anchor, size, seal), which, according to IFU, should be checked by TEE, albeit, in principle, it is possible to perform this evaluation based on fluoroscopy also.

Against this background, the assumption is justified that, nowadays, LAAC might be performed safely under sole fluoroscopic guidance if dedicated CT-based preplanning of the procedure is performed. This hypothesis is based on the existing clinical experience regarding the technique of left atrial appendage closure and the technical improvements that have been implemented in the development of new-generation LAAC systems.

## 2. Methods

Fluoro-FLX was a prospective, single-center trial performed at Catholic Hospital “St. Johann Nepomuk” Erfurt, Germany; ideas regarding study design were adopted from [6]. The trial was approved by the ethics committee of the medical association of Thuringia and is registered at the German Registry of Clinical Trials, DRKS00023464. Consecutive patients that were planned for LAAC were screened for study inclusion. Inclusion criteria were age >18 years, atrial fibrillation (paroxysmal, persistent, or permanent) with planned interventional left atrial appendage closure using the WATCHMAN FLX, signed informed consent, exclusion of intracardiac thrombi by cardiac CT within 72 h before LAAC, and no long-term interruption of therapeutic anticoagulation before the start of LAAC (i.e., last administration of NOAC or LMW heparin within 48 h before LAAC). Exclusion criteria were a history of ASD/PFO closure, contraindication regarding TEE, lack of informed consent, and anticipated inability to perform a 3-month follow-up (including TEE).

After obtaining informed consent, CT images (64-slice, Somatom Perspective, Siemens Healthcare, Erlangen, Germany) were analyzed using 3D LEONARDO Workstation (Siemens Healthcare) (i) to exclude intracardiac thrombi, (ii) to predict two optimal C-arm angulations with an orthogonal projection of the LAA ostium with a distance of at least 60°, (iii) to measure the diameter of the LAA landing zone. During LAAC, all patients were sedated by continuous IV administration of propofol 1%, with the addition of fentanyl as required. The TEE probe was inserted at the beginning of the procedure, and the monitor of the ultrasound device was arranged in a way that it could only be seen by the echo physician but not by the interventionalist. After puncturing the right femoral vein, a standardized transseptal puncture (TSP) was prepared with sole fluoroscopic guidance. When the transseptal needle was in place, confirmation of a safe puncture site was confirmed by the echo physician (checkpoint 1) before TSP was performed. Anticoagulation was ensured by IV administration of unfractionated heparin with a target ACT of 250–300 s. After TSP, an Amplatz Super Stiff wire (Boston Scientific, Marlborough, MA, USA) was placed in a left pulmonary vein, and the transseptal TruSeal sheath (Boston Scientific, Marlborough, MA, USA) was advanced into the left atrium (LA). A pigtail catheter was then used to obtain angiography of the LAA at least in one of the two CT-defined angiographic projections. The dimensions of the LAA were determined by the interventionalist by quantification of the angiographic visualization of the LAA and compared with the measurements that had been obtained by CT. Afterward, the selection of the appropriate size of the WM FLX (Boston Scientific, Marlborough, MA, USA) was made by the interventionalist and documented and communicated to the echo physician. The echo physician had to confirm the correct device sizing (checkpoint 2), then the device packaging was opened, and the WM FLX was prepared and stepwise placed into the LAA according to standard techniques. Adequate device compression and the proper position in the LAA ostium, as well as the absence of a para-device leak, were determined at both of the 2 angiographic projections that had been selected previously on the basis of the CT preplanning. If, according to the opinion of the interventionalist, all release criteria were met, the echo physician had to confirm the correct device position (checkpoint 3), and the occluder was then released from the catheter. At any time during the procedure, the echo physician could interrupt the procedure if complications or safety-relevant abnormalities would occur. Any disclosure of the TEE to the interventionalist was recorded in the study documentation. Follow-up TEE was carried out 3 months after LAAC.

For calculation of device compression, the diameter of the device was quantified either in angiography or in TEE (0°, 45°, 90°, 135°), respectively, and calculated as compression [%] = 100% − 100 × [(real device size − measured size)/real device size].

A preprint of this study has previously been published [8].

### Study Endpoint

The primary endpoint of this study was defined as the percentage of LAAC procedures without interruption by TEE. For statistical analysis, a performance goal was defined based on a presumed efficacy of LAAC of 97.5% and a margin of equivalence of 8%, leading to a sample size of 30 patients at a power of ß = 0.80.

## 3. Results

**Figure 1:**
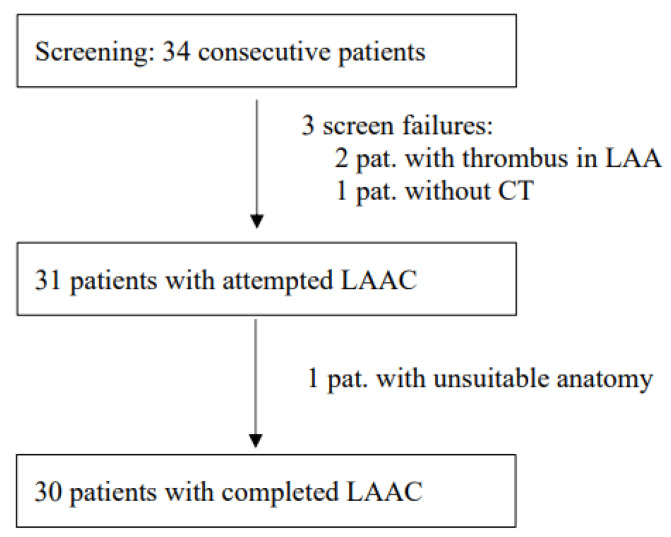
Patient flow. (LAA: left atrial appendage; CT: computed tomography; LAAC: left atrial appendage closure; pat.: patient).

Between 28 October 2020 and 20 May 2021, 34 consecutive patients were screened for study inclusion (Figure 1). All CT datasets were suitable for assessing the anatomy of the LAA and performing LAAC preplanning. In 2 cases, thrombi could not be definitely ruled out with CT due to poor contrast penetration into the LAA, so a pre-implantation TEE using an echo contrast agent (SonoVue) was performed additionally, as described before [9]. In 31 patients, LAAC was attempted, and in 30 patients, LAAC could be technically completed. In one patient, no adequate positioning of the WM FLX device could be achieved (reversed chicken wing anatomy). In that case, a conventional approach using standard TEE guiding was undertaken directly after the study procedure, which also did not lead to technical success.

**Figure 2:**
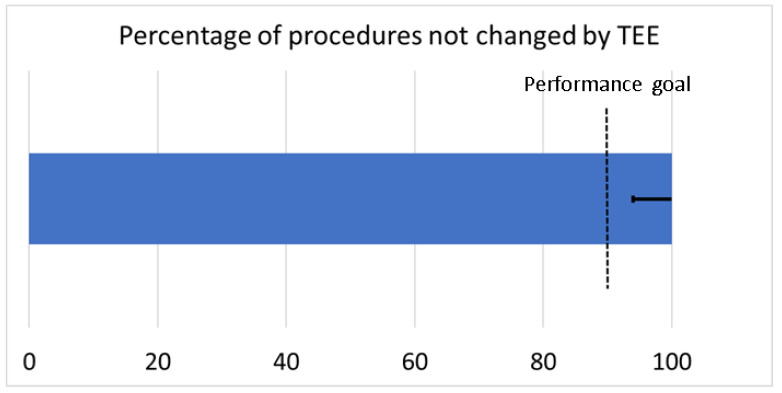
Percentage of LAAC procedures that have not been influenced by TEE findings (N = 31). The error bar shows the 95% confidence interval (Likelihood); it does not cross the specified performance goal, so the primary endpoint is met. (LAAC: left atrial appendage closure; TEE: transesophageal echocardiography).

Demographic and clinical data of the patients are given in Table 1; procedural characteristics of the LAA closure and implantation results after the release of the WM FLX, as well as at the 3-month follow-up, are given in Table 2. In none of the cases did TEE lead to an interruption or change in the implantation procedure, thereby reaching the primary study endpoint (Figure 2).

During this study, it became evident that the renunciation of TEE led to a slight increase in contrast medium in comparison to previous experiences from our center [10]. Therefore, after 20 patients, we started to apply biplanar fluoroscopy, which led to a reduction in contrast exposure, as expected (monoplane: 77 ± 26 mL; biplane: 55 ± 21 mL; *p* = 0.02), with no significant change in radiation dose (monoplane: 19 ± 11 Gy × cm^2^; biplane: 24 ± 20 Gy × cm^2^; *p* = 0.45) or procedure time (monoplane: 33 ± 7 min; biplane: 30 ± 10 min; *p* = 0.38), respectively.

As seen in Table 3, no adverse cardiac or cerebrovascular clinical endpoints were observed during this study. However, in one patient, an adverse event related to the TEE was documented (pharyngeal bleeding).

## 4. Discussion

LAAC is, nowadays, a proven alternative for patients with atrial fibrillation and a high thromboembolic risk who are not considered suitable for oral anticoagulation [11,12]. Since the early days of this technology, a number of improvements have been implemented into new devices and interventional strategies, including imaging, so the procedure of LAAC has become both more effective and safer [11,13]. Today, the vast majority of cases are still performed under the guidance of TEE, although this contains several drawbacks deriving from the need for patient sedation and the semi-invasive nature of the TEE [3,4]. Our study presented here shows that, in principle, it is feasible to restrain from TEE if a dedicated CT-based planning algorithm of the procedure and the newest generation of the LAA closure device are used.

So far, there is only limited evidence regarding the question of to what extent TEE guidance indeed has an influence on the procedure of LAAC and whether echo guidance might perhaps be dispensable [14,15]. Recently, a paper was published describing the experience of a single high-volume center where 811 LAAC procedures were performed, either with (N = 262) or without TEE guidance (N = 549), between 2009 and 2020 [16]. In this retrospective analysis, the echo-guided approach was linked to favorable efficacy and safety outcomes. However, no CT-based procedure planning was used in these cases. Additionally, in the group with sole fluoroscopic guidance, the newest generation of LAA closure device, WATCHMAN FLX, was not used at all, so these findings should not be compared directly to our data.

In our study, we have implemented a dedicated CT-planning algorithm in order to increase the accuracy and success rate of a fluoroscopy-only-based LAAC. To achieve an optimal implantation result and, especially, to avoid device embolization, the WM FLX should be positioned at, or just distal to, the LAA ostium. This might be difficult to evaluate with fluoroscopy if the angulation of the C-arm is not aligned perpendicular to the LAA ostial plane. In our study, we have overcome this drawback by using cardiac CT to predict patient-specific optimal C-arm angulations that were used during the intervention. We have shown previously that an optimal selection of angiographic views positively impacts LAAC [10]. Additionally, it is important to ensure that all lobes of the LAA are finally covered and, thereby, sealed by the LAA closure device so that there is no relevant residual paradevice leakage (≤5 mm; [17]). Assuming that a paradevice contrast leak is safely identified by fluoroscopy if the contrast medium exceeds the metal border of the WM FLX by at least 1.5 mm, all leaks larger than 3 mm can be identified if two angiographic views are checked with a distance in angulation of at least 60° (e.g., RAO 30/LAO 30). Again, the CT-based prediction of the patient-specific optimal C-arm angulations allows this leak detection with high accuracy.

## 5. Study Limitations

Our study consists of only 31 LAAC cases, which limits the statistical strength, especially regarding clinical outcomes and complications such as LAA perforation, device embolization, or ischemic stroke, which do not occur frequently. Additionally, a 3-month follow-up TEE was available only in 23 patients, which was mainly caused by the ongoing COVID-19 pandemic. According to the study protocol, only procedures with the WM FLX were included in the trial, so no comparison between different LAAC devices can be given. It should be emphasized that all procedures were carried out by an expert LAAC team, so the results might not be generalized to less experienced operators.

## 6. Conclusions

Our prospective single-center study shows that in the majority of cases, the diagnostic yield of TEE during LAAC is rather limited, so TEE might be considered dispensable if a dedicated CT-based preplanning of the procedure is performed and the newest generation of LAA closure devices is used. Especially in patients who are at high risk for TEE-related adverse events, this might be worth considering for experienced operators.

## Figures and Tables

**Table 1:** Baseline demographic, clinical characteristics, and CT-derived parameters of patients undergoing LAA closure (N = 31). (LAA: left atrial appendage; eGFR: estimated glomerular filtration rate).

Parameter	Value (%)
**demographics**	
female sex	13 (45%)
age (years)	78.9 ± 7.3
height (cm)	171 ± 11
weight (kg)	84 ± 20
hypertension	28 (93%)
diabetes	15 (50%)
history of stroke	8 (27%)
coronary artery disease	9 (30%)
CHA_2_DS_2_VASc	4.6 ± 1.4
HASBLED	3.2 ± 1.0
eGFR (mL/min × 1.73 m^2^)	52 ± 21
**AF pattern**	
paroxysmal	10 (33%)
persistent	4 (13%)
permanent	16 (53%)
**baseline CT**	**value**
max. diameter of LAA landing zone (mm)	25.4 ± 3.5
predicted C-arm angulation	
first projection	−29.0 ± 8.7/0.3 ± 14.2
second projection	35.6 ± 10.5/−30.5 ± 9.4

**Table 2:** Procedural characteristics of LAA closure and implantation results. All procedures were performed using the WATCHMAN FLX device. Device compression was calculated as compression [%] = 100% − 100 × [(real device size − measured size)/real device size]. (TSP: transseptal puncture; LAA: left atrial appendage).

Parameter	Value
technical implant success	30/31 (96.8%)
procedure time	32 ± 8 min
time from venous access to	
TSP	7 ± 5 min
LAA angiography	17 ± 7 min
opening of device package	19 ± 7 min
final placement of device according to fluoroscopy	29 ± 8 min
release of device	30 ± 8 min
access site closure	32 ± 8 min
size of LAA in angiography (mm)	24.0 ± 3.3
contrast medium (ml)	70 ± 26
radiation (Gy × cm^2^)	20.8 ± 14.2
number of devices used	1.0 ± 0.0
**size of implanted device (mm)**	
20	0 (0%)
24	6 (20%)
27	8 (27%)
31	12 (40%)
35	4 (13%)
**TEE measurements after device release (N = 30)**	
compression (%)	
minimum	13.0 ± 5.8
maximum	20.7 ± 5.9
mean	16.9 ± 5.5
maximum leak	
no	29 (97%)
<3 mm	1 (3%)
3 to 5 mm	0 (0%)
>5 mm	0 (0%)
max. protrusion towards LA (mm)	
minimum	2.0 ± 3.0
maximum	9.7 ± 3.7
mean	6.0 ± 3.1
interruption or change in interventional procedure due to TEE finding	0 (0%)
adverse event due to TEE	1 (3%)
**TEE measurements after 3 months (N = 23)**	
compression (%)	
minimum	11.5 ± 6.9
maximum	20.5 ± 7.5
mean	16.0 ± 6.4
maximum leak	
no	20 (87%)
<3 mm	2 (9%)
3 to 5 mm	1 (4%)
>5 mm	0 (0%)
maximal protrusion towards LA (mm)	
minimum	2.4 ± 3.7
maximum	8.9 ± 4.1
mean	5.7 ± 3.7

**Table 3:** Clinical endpoints after LAAC until hospital discharge and given medication. (TIA: transient ischaemic attack; DAPT: dual antiplatelet therapy; VKA: Vitamin K antagonist; NOAC: Non-Vitamin K antagonist oral anticoagulants).

Clinical Endpoint	Value
pericardial efflusion	0 (0%)
TIA	0 (0%)
stroke	0 (0%)
systemic embolism	0 (0%)
device embolism	0 (0%)
death	0 (0%)
medication	
aspirin	29 (97%)
clopidogrel	28 (93%)
ticagrelor	0 (0%)
prasugrel	0 (0%)
DAPT	27 (90%)
VKA	0 (0%)
NOAC	3 (10%)
NOAC + antiplatelet	2 (7%)

## Data Availability

Data can be obtained from the authors on justified request.

## Abbreviations

LAA	left atrial appendage
LAAC	left atrial appendage closure
AF	atrial fibrillation
CT	computed tomography
TEE	transesophageal echocardiography
OAC	oral anticoagulation
NOAC	non vitamin K antagonist oral anticoagulants
TSP	transseptal puncture
WM	WATCHMAN
